# Three-Dimensional Models: Biomimetic Tools That Recapitulate Breast Tissue Architecture and Microenvironment to Study Ductal Carcinoma In Situ Transition to Invasive Ductal Breast Cancer

**DOI:** 10.3390/cells14030220

**Published:** 2025-02-04

**Authors:** Seema Shah, Kingsley O. Osuala, Ethan J. Brock, Kyungmin Ji, Bonnie F. Sloane, Raymond R. Mattingly

**Affiliations:** 1 Department of Oncology, Wayne State University School of Medicine, Detroit, MI 48201, USA; bo4026@wayne.edu (S.S.); ebrock@med.wayne.edu (E.J.B.);; 2 Twelve Biosciences, Kalamazoo, MI 49009, USA; ey0453@wayne.edu; 3 Department of Neurology, Henry Ford Health, Detroit, MI 48202, USA; 4 Department of Pharmacology, Wayne State University School of Medicine, Detroit, MI 48201, USA; 5 Department of Pharmacology and Toxicology, Brody School of Medicine, East Carolina University, Greenville, NC 27834, USA

**Keywords:** DCIS, IDC, microfluidics, 3D cell culture, tumor microenvironment, extracellular matrix

## Abstract

Diagnosis of ductal carcinoma in situ (DCIS) presents a challenge as we cannot yet distinguish between those lesions that remain dormant from cases that may progress to invasive ductal breast cancer (IDC) and require therapeutic intervention. Our overall interest is to develop biomimetic three-dimensional (3D) models that more accurately recapitulate the structure and characteristics of pre-invasive breast cancer in order to study the underlying mechanisms driving malignant progression. These models allow us to mimic the microenvironment to investigate many aspects of mammary cell biology, including the role of the extracellular matrix (ECM), the interaction between carcinoma-associated fibroblasts (CAFs) and epithelial cells, and the dynamics of cytoskeletal reorganization. In this review article, we outline the significance of 3D culture models as reliable pre-clinical tools that mimic the in vivo tumor microenvironment and facilitate the study of DCIS lesions as they progress to invasive breast cancer. We also discuss the role of CAFs and other stromal cells in DCIS transition as well as the clinical significance of emerging technologies like tumor-on-chip and co-culture models.

## 1. Introduction

Ductal carcinoma in situ (DCIS) accounts for under a quarter of newly diagnosed cases of breast cancer in the United States [1,2]. The rise in breast cancer incidence for women between the 1970s and 2015 has largely been attributed to the increased use of mammographic screening methods to detect lesions—including DCIS—earlier. Unfortunately, despite the increased detection and treatment of DCIS, there has not been a concurrent decrease in invasive ductal cancer (IDC) [1]. Ultimately, this is both a technological and a biological issue. On the imaging side, increased screening sensitivity has translated to higher rates of false positives [3]. However, the application of existing technology continues to be refined while novel methods undergo rigorous examination with the goal of achieving higher rates of sensitivity, specificity, and accuracy [4].

Currently, as many as one-third of breast lesions (including DCIS) identified in asymptomatic women via routine mammography may fit the description of being over diagnosed [5]. One reason for this is that we cannot yet distinguish between DCIS lesions that will remain dormant and those that will progress to IDC and require therapeutic intervention. Additionally, there is still much to understand in terms of how DCIS progresses to IDC due to its heterogeneous course in evolution [5]. This heterogeneity is not only observed between patients but also within an individual [6]. Due to this uncertainty, treatment of DCIS has historically been extrapolated from studies on invasive breast cancer [7]. As a result, many low-risk DCIS lesions are likely subject to excessive treatment. This potential for overtreatment has generated much discussion around the negative financial, physical, and mental impacts on a patient’s life [8,9,10]. Patients with breast cancer worldwide are at risk of financial toxicity (cancer-related financial distress). A systematic review found that 78.8% of patients in low- and middle-income countries as well as 35.3% of patients in high-income countries experienced financial toxicity with regard to breast cancer treatment [11]. As of 2020, this translated to a cost of over USD 500 million for the primary management of all DCIS cases diagnosed in the United States [12]. Furthermore, breast cancer patients paid higher out of pocket costs for their care (USD 3.14 billion) than patients with prostate, colorectal, and lung cancers (USD 2.26, USD 1.46, and USD 1.35 billion, respectively) in 2019 [13]. In other words, there is a clear need for additional research and modified treatment protocols to mitigate overtreatment without compromising therapeutic outcomes. This would spare women with low-risk DCIS from the exposure of intensive treatments, preserve their quality of life, and provide a reprieve from the associated financial burdens brought on by unnecessary treatments [3].

At present, there are no validated prognostic or predictive multigene signatures that can successfully identify DCIS patients (1) who are at risk of invasive recurrence, (2) whose risk is not further reduced by radiation therapy, and (3) whose lesions warrant no adjuvant therapy [14]. However, these are incredibly stringent criteria to meet collectively. Substantial progress has been made over the past decade toward this goal in the form of both the Oncotype DX Breast DCIS Score and DCISionRT. The Oncotype DX Breast DCIS Score—a subset of 12 genes from the Oncotype DX Recurrence Score—is a clinically validated, commercially available, multigene prognostic assay that can be used to quantify the 10-year risk of local or invasive recurrence after a diagnosis of DCIS without the addition of radiation therapy [15,16]. It was shown in the DUCHESS prospective cohort study that the DCIS Score led to a change in treatment recommendations for 35% of patients and that its combination with clinicopathological data decreased radiation therapy recommendations from 79% to 50% compared to clinicopathological data alone [17]. Its inclusion was also associated with a reduction in decision-based conflict and better patient satisfaction, aligning with results from a previous study [17,18].

Similar to the Oncotype DX Breast DCIS Score, DCISionRT is a clinically validated and commercially available multigene prognostic assay that can be used to quantify the 10-year risk of local or invasive recurrence [19,20,21,22,23,24]. However, it differs notably in its ability to predict patient benefit from radiotherapy [19,20,21]. Leveraging machine learning, Bremer and colleagues ultimately arrived at a seven-gene signature that integrated clinicopathologic factors and biologic pathway data to generate a “decision score” [19]. This score was then used to reclassify patients into low- and elevated-risk groups. While the low-risk group did not appear to benefit from radiation therapy, the group marked as having an increased risk by the decision score received substantial benefit over the baseline [19,20,23,24]. Recently published data from Part I of the PREDICT trial showed that DCISionRT results changed radiation therapy recommendations in 38% of cases [24]. Of the 1424 patients initially recommended adjuvant radiotherapy, 1024 maintained that recommendation post-DCISionRT result (a 20% reduction). Part II of the trial will seek to recruit an additional 3000 individuals and will be conducted in the same manner as Part I, with the following exceptions: (1) it will use an updated test protocol to classify patients into three risk groups (low, elevated, and residual) instead of low or elevated, (2) it will collect more in-depth data about patients, and (3) it will delve into how physicians utilize the residual risk group to make treatment decisions [25].

As of this writing, neither the Oncotype DX Breast DCIS Score nor DCISionRT are widely incorporated into standard practice. However, with their continued use and validation, broader clinical use may be on the horizon. These tests represent an important step toward refining the treatment of DCIS and improving patient quality of life (provided that their implementation is not cost-prohibitive) [16,26,27,28]. In the meantime, much can be learned from clinical trials that are currently underway.

It is estimated that 90% of the changes in stromal cancer biology, such as extracellular matrix (ECM) remodeling, gene expression, and chemical cues (hormones, cytokines, and growth factors), occur at the DCIS stage [29] and correlate with patient outcome [30]. Thus, the problem of overdiagnosis, coupled with the complex nature of DCIS progression, underscores the need to better elucidate such biomarkers and molecular pathways to facilitate more targeted treatment options. Given these challenges, PRECISION (PREvent ductal Carcinoma In Situ Invasive Overtreatment Now), was formed (https://www.dcisprecision.org) (accessed on 3 December 2024). This international consortium is built on the COMET (NCT02926911; US), LORD (NCT02492607; Netherlands/EU), and LORIS (ISRCTN: 27544579; UK) clinical trials. The goal of these studies is to determine the feasibility of active surveillance (compared to standard care) in managing low-risk DCIS for women (age 40+). The COMET trial recently found that active monitoring did not have a higher rate of invasive cancer compared to standard care [27]. Additionally, no difference was observed between groups regarding their lived experience (e.g., anxiety, depression, overall quality of life) [31]. However, it should be noted that these results are limited by a short observation window. Analyses of the cohort at 5, 7, and 10 years will likely provide a more realistic view of any differences between the groups. LORD is still actively recruiting, though an intermediate report noted a strong preference for the active surveillance arm (288/377) compared to standard care (89/377) [32]. The LORIS trial has closed early due to the limited availability of eligible patients and patient preference [33]. Of the 227 eligible women, 183 agreed to be randomized, though two had to withdraw due to COVID-related issues. The remaining 181 women were assigned to one of the two arms, and we await the 10-year results. Using these trials, PRECISION aims to reduce the burden of the surgery, radiation, and hormone-blocking therapies used in the management of DCIS without compromising outcomes—thereby reducing overtreatment. This will be achieved using established and innovative methods to analyze DCIS for the purpose of pinpointing characteristics that differentiate between aggressive and indolent lesions. This complements the necessity to develop biomimetic in vitro 3D models that accurately recapitulate the heterogeneity of the disease, as well as the characteristics and influence of the tissue microenvironment. As we continue in this review, we will discuss the various cell types found in the breast cancer microenvironment and examine several 3D in vitro breast cancer modeling systems, including our TAME (Tissue Architecture and Microenvironment Engineering) 3D cell culture platform.

## 2. The DCIS Microenvironment

The tumor microenvironment (TME) is a complex ecosystem that is home to many non-tumor cells that have acquired an altered phenotype [34]. In this niche, tumor cells communicate with neighboring cells via matrix proteins, growth factors, cytokines, etc. [35,36], and it is well established that the microenvironment provides signals to both tumor and non-tumor cells [37]. Previous efforts to identify predictive signatures have focused on comparing tumor cells in DCIS to those in IDC and have unfortunately yielded limited information, reporting no significant differences between the transcriptomic signature of neoplastic cells throughout disease progression [38]. With the lack of distinct genetic changes between DCIS and IDC, the hypothesis that the potent drivers of DCIS progression are present in the microenvironment has emerged. This hypothesis has been supported by reports that the transition to IDC requires changes in the stroma where a tumor-promoting niche facilitates local invasion [39,40]. In particular, the Breast PreCancer Atlas, a subproject of the Human Tumor Atlas Network, reported that patient-matched DCIS and IDC revealed coordinated changes in the TME associated with invasive relapse [40]. This finding supports the role of the TME as a major contributor to invasion. Thus, efforts for mining the TME are gaining significant traction.

### 2.1. The Biological Significance of the ECM and Its Role in DCIS Transition

The ECM, a component of the DCIS TME, is a complex network of macromolecules. It is a highly dynamic structure, subject to carefully orchestrated spatiotemporal rearrangement, geometric patterning, and remodeling by various factors, including proteases [41]. It is composed of a basement membrane (BM) and an interstitial matrix [42]. Where the BM is made up of type IV collagens, laminin, fibronectin, and linker proteins such as entactin, the interstitial matrix is rich in fibrillar collagen, glycoproteins (notably proteoglycans), and fibronectin [43]. The components of the ECM are secreted and arranged by cells according to the dynamic needs of the tissue. For example, in the human breast, the ducts—consisting of luminal and myoepithelial cells—are encased by BM, which separates the epithelial cells from the stroma. Without this compartmentalization, the tissue’s normal physiological functions would be disrupted.

Collagen is the most abundant component in the breast microenvironment and its organization in breast tissue contributes to the mechanical properties of the matrix [44]. Breast epithelial cells mechano-sense their microenvironment through collagen binding receptors such as discoidin domain receptors [45] and integrin receptors [46]. In DCIS and other breast cancers, the ECM of the mammary gland becomes progressively stiffer and collagen-rich compared with healthy breast tissue. Extracellular matrix stiffness is a result of excess collagen deposition and cross-linking. Discoidin domain receptors on the epithelial cell surface bind to collagen, which in turn triggers their nuclear translocation and activation of the epithelial–mesenchymal transition (EMT) transcriptional program [47]. Furthermore, a collagen-rich stiff matrix is associated with the nuclear translocation of TWIST1 (a marker of EMT) in a β-integrin-dependent manner [48]. In addition to increased collagen deposition and crosslinking, the orientation of collagen fibers is altered in disease progression. Collagen fiber alignment profiles have been well characterized in breast cancer and are referred to as tumor-associated-collagen signatures (TACS) [49]. TACS-1 is associated with increased collagen fiber density surrounding the tumor; TACS-2 is subsequent re-alignment of collagen fibers parallel to the tumor border; TACS-3 is collagen realignment perpendicular to the tumor border and is associated with invasion [50,51]. These changes are correlated with tumor grade and poorer patient outcome [52]. In addition, collagen fibers are often observed to be oriented perpendicular to the duct in DCIS exhibiting poor prognostic markers, such as the absence of estrogen and progesterone receptor (ER/PR) expression and the presence of necrosis [53].

Alterations of other matrix components are also implicated in promoting early breast cancer progression. For example, the deposition of periductal fibronectin by myoepithelial cells is increased in DCIS as compared to healthy ducts and is thought to support a pro-invasive niche [54]. This profound influence of the ECM in the mammary gland continues to enable new lines of inquiry, including “-omics” or bioinformatic approaches, to gain further functional insights. To this end, a protein knowledge database known as MatrisomeDB has been constructed by compiling proteomics data on the ECM composition of different tissues and tumor types [55]. MatrisomeDB currently includes 847 human and 791 mouse ECM proteoforms and over 350,000 human and mouse ECM-derived peptide-to-spectrum matches [56]. Specific to breast cancer, temporal profiling of the matrisome (which consists of genes encoding ECM- and ECM-associated proteins) has revealed collagen XII as a key component in the regulation of collagen I organization, with increased secretion associated with poor prognosis [57].

### 2.2. CAFs and Their Role in DCIS Progression

In normal, healthy tissue the stroma is populated by fibroblasts [58], which are typically found near ductal structures [38]. Fibroblasts are commonly quiescent under homeostatic conditions. However, during the various stages of mammary gland development and pregnancy they provide structural support through the secretion of matrix proteins and promote epithelial growth and differentiation. In addition, fibroblasts support stromal organization during branching morphogenesis via the secretion of proteases such as Matrix metalloproteinase 3 (MMP-3) [59]. The fine-tuned activation of proteases also plays a role in the involution of the mammary gland [60]. Thus, fibroblasts play an outsized role in maintaining tissue homeostasis.

Under abnormal proliferative conditions, pathologically activated fibroblasts—also known as carcinoma-associated fibroblasts (CAFs)—account for the most abundant stromal cell types [61,62] and are, for the most part, derived from resident fibroblasts that have acquire a myoblast-like phenotype [63]. They have been shown to be pivotal players in breast tumor initiation and progression [61,64,65,66] and may represent up to 70% of a breast tumor’s mass, though the fraction of CAFs present varies from patient to patient [67]. Additionally, up to 80% of normal fibroblasts in breast tissue may acquire a CAF phenotype during breast cancer progression [68,69]. A recent study [70] performed single-cell RNA sequencing of 768 transcriptomes of mesenchymal cells derived from a genetically engineered mouse model of breast cancer and identified three distinct subpopulations of CAFs. The genetic profiles of two subpopulations were found to correspond to distinct functional programs and held independent prognostic value in clinical cohorts by association with metastatic disease. These CAF subpopulations also represent an important component of DCIS heterogeneity.

As previously mentioned, the mammary gland becomes progressively stiffer and collagen-rich in breast cancer compared with healthy tissue. These changes in matrix remodeling are predominantly attributed to the action of CAFs [38]. This is further strengthened by the finding that CAFs play a role in dysregulating collagen turnover, leading to desmoplasia and excessive collagen deposition [71]. The excessive stiffening of the ECM and secretion of MMP enzymes create a self-sustaining positive feedback loop of CAF activation and ECM remodeling, resulting in biomechanical and biochemical changes that foster tumor growth and invasion [72].

In vivo studies have shown that co-injecting tumor cells and fibroblasts leads to the increased formation of invasive tumors compared to tumor cells alone [73]. In vitro 3D co-culture studies with CAFs and MCF10.DCIS cells reinforce this finding, showing that CAFs promote the invasive capability of cells by increasing MMP-14 and -19 levels, changing collagen organization, and increasing proliferation rate [74,75]. In basal-like DCIS, the reorientation of collagen fibers by CAFs contributes to invasive potential [76] and is characterized by perpendicular alignment near invasive ductal cancers [51,76]. Conversely, in vitro 3D culture studies from our lab [36] and others [77] have shown that normal fibroblasts regulate the phenotypic behavior of breast ductal cells by reverting DCIS cells, and in some cases invasive cancer cells, to a more normal phenotype.

### 2.3. Other Stromal Cells in DCIS Progression

#### 2.3.1. Myoepithelial Cells

Myoepithelial cells, located between the BM and breast epithelial cells, are best known for their role in the contraction of the mammary ducts during lactation [78]. They act as guardians of polarity, ensuring luminal and ductal cells do not cross into the surrounding stroma [38], and exhibit anti-proliferative and anti-invasive properties. This can be seen in a time-lapse study of 3D organoids where myoepithelial cells restrained and recaptured invasive luminal cells [79]. Three-dimensional co-culture studies show that myoepithelial cell interactions suppress tumor cell invasion, reverting tumor growth to a less invasive, DCIS-like state [80]. Myoepithelial cells contribute to the synthesis and organization of the BM through the secretion of laminin and collagen IV, and also help regulate the differentiation of luminal cells [81]. With regard to DCIS, myoepithelial cells have been shown to line the ducts as in normal conditions; however, they show a reduction of laminin-1 expression, which has been correlated to an inability to properly polarize luminal epithelial cells [82]. Additionally, myoepithelial cells showing increased expression of *CXCL12* and *CXCL14* had a tumor-promoting role, demonstrating enhanced cancer cell proliferation and migration [83]. In support of the anti-tumor role of normal myoepithelial cells, we have shown that they reduce the dysplastic phenotype of DCIS and inhibit CAF-induced ECM proteolysis and invasion [84].

#### 2.3.2. Adipocytes

Adipocytes, long considered as energy stores, also play a role in tumor growth. Now termed cancer-associated adipocytes (CAAs), there is increasing emphasis on these cells in the DCIS tumor microenvironment. Xenograft and in vitro studies have shown that the interaction between DCIS cells and CAAs is reciprocal. DCIS cells block the adipogenic differentiation of pre-adipocytes while inducing the expression of fibroblast-secreted protein-1 and α-smooth muscle actin, which are usually associated with CAFs. In turn, CAAs facilitate the proliferation and invasion of cancer cells both in vitro and in xenograft models [85]. The adipose compartment has also been reported to be an inhibitor of myoepithelial cell function via the reduction of laminin and collagen IV secretion [86]. In addition, preadipocyte-derived exosomes have been shown to promote a stem cell phenotype in DCIS [87].

A major limitation of previous in vitro studies was the use of 2D adipocyte cultures. However, Shen and colleagues recently demonstrated a 3D adipose spheroid culture model that yielded adipocytes that more closely resembled in vivo morphology, phenotype, and genotype [88]. Additionally, their model remained phenotypically stable in 3D culture for up to 6 weeks. Such a model increases the physiological relevance of adipose studies and is a step closer to building organotypic in vitro models that reflect the constituents of in vivo DCIS biology.

#### 2.3.3. Tumor-Infiltrating Lymphocytes

Through the process of immunoediting, cancer cells are known to disrupt the immune system’s ability to properly activate checkpoint pathways, creating an immunosuppressive microenvironment [89]. In the pre-invasive context, patient cohort data suggest that DCIS-associated stromal changes may reduce the interaction between immune cells and DCIS cells in a subtype-dependent manner. Immunohistochemical analysis of stromal tissues showed that fewer immune cells were present in DCIS with stromal changes compared to those without, and that intraductal clusters of differentiation (CD)8+ lymphocytes were most common in human epidermal growth factor receptor-2 positive (HER2+)/ER+ DCIS compared to other subtypes [90]. Conversely, a retrospective study by Kim and colleagues found that DCIS tended to have more CD4+ than CD8+ tumor-infiltrating lymphocytes (TILs), regardless of hormone receptor status [91]. This discrepancy may be explained by differences in sample categorization, as the latter work did not stratify their results based on HER2 status. Regardless, work by both groups implies that higher infiltration of regulatory T-cells fosters an immunosuppressive microenvironment, and that CD4+, CD8+, and FOXP3+ lymphocytes, as well as the presence of PD-L1+ immune cells, are associated with high nuclear grade, comedo necrosis, hormone receptor negativity, and high Ki-67 proliferation index [90,91,92]. This corroborates previous work that showed higher levels of tumor-infiltrating cells are present in high-grade ER-/HER2+ DCIS [93] and that high tumor-infiltrating cell density is a significant predictor of ipsilateral recurrence in DCIS [94,95].

In February of 2024, the US Food & Drug Administration approved Lifileucel (Amtagvi, Iovance Biotherapeutics, Inc., San Carlos, CA, USA ), a novel TIL-based therapy to treat unresectable or metastatic melanoma in patients who had previously received a PD-1 blocking antibody or BRAF inhibitor (with or without a MEK inhibitor if BRAF V600+) [96]. This therapy utilizes a patient’s own TILs to better fight cancer cells. Briefly, a biopsy is collected from the patient, where TILs are extracted and cultured to expand their numbers into the billions (see Tran et al. for a most common protocol) [97]. These expanded TILs may or may not be genetically modified to enhance their cancer cell recognition. Upon expansion, TILs are returned to the patient via infusion after a short-term chemotherapy to prepare the patients’ immune system for the influx of TILs [98].

#### 2.3.4. Tumor-Associated Macrophages

Tumor-associated macrophages (TAMs) represent a significant component of tumor-infiltrating immune cells in breast cancer [99]; they can be classified as pro-inflammatory (M1) or anti-inflammatory (M2) [100]. Tumor-derived growth factors such as chemokines and cytokines facilitate the recruitment of monocytes and macrophages into the immediate TME [101], and it is this environment that regulates the differentiation and polarization of TAMs into M1 or M2 [102]. In vitro and in vivo studies have shown that macrophages are involved in a feedback loop between tumor cells at invasive fronts in breast tumors, where they enhance tumorigenesis [102]. TAMs promote angiogenesis and produce MMP-7 and -9, which facilitate basement membrane dissolution and tumor escape [103]. With regard to DCIS, cohort studies show that TAMs, regardless of type, are associated with early ipsilateral recurrence [104]. Tissues with a dense population of TAMs potentially serve as predictors of DCIS microinvasion.

## 3. Significance of 3D Models

Traditional monolayer culture systems have long been the gold standard for the study of cellular dynamics such as changes in cell–cell adhesion, cell–cell interaction, and changes in actin-cytoskeletal remodeling and the mechanisms behind them, as well as drug efficacy. However, they do not reflect the in vivo environment as they lack a dynamic and living ECM, which contains key natural and functional cues that govern fundamental aspects of cell biology [105], including signaling [106] and drug sensitivity [107,108]. The significance of 3D organoid cultures as tools to catalog and characterize the heterogeneity of breast cancer is underscored by the establishment of over 100 primary and metastatic breast cancer organoid cell lines (including DCIS) that can be subject to downstream analyses and drug discovery [109].

In vivo mammary gland mouse models are pivotal to studying many aspects of breast biology, especially breast cancer progression. In 2009, Behbod and colleagues published their methodology for the Mouse INtraDuctal (MIND) human-in-mouse transplantation model [110]. This in vivo system is particularly relevant because it more closely models DCIS compared to previously established models that usually involve injection into a cleared and humanized mammary fat pad or subcutaneous injection. Their intraductal model was established by injecting human DCIS cell lines MCF10.DCIS.com, SUM 225, and FSK-H7 (derived from primary human DCIS) directly into the mouse duct via a cleaved nipple. Additional work from the same group showed that this successfully mimicked some of the diversity observed in pre-invasive DCIS in vivo and that the technique was able to preserve and mimic histological subtypes such as micropapillary, papillary, cribriform, solid, and comedo DCIS. The molecular characteristics were classified according to the expression of human cytokeratins and ER and HER2 status [111]. More importantly, this work showed that MIND models mimic clonal heterogeneity of human DCIS [112]. Additionally, Hutten and colleagues [113] generated a living biobank of 115 DCIS-MIND models. These MIND models were characterized by grade, HER2 amplification, copy number alterations, and expansive 3D growth. They also showed that sequential transplantation of xenografts showed minimal phenotypic and genotypic changes over time, supporting a clonal evolution model [113].

While useful, in vivo models are expensive and require a significant amount of time, effort, technical skill, and logistics to maintain. Additionally, mouse mammary gland physiology does not accurately mimic human breast physiology [114]. While human breast tissue in general is more fibrous, mouse models contain more adipose tissue [115]. Three-dimensional culture models provide a line of inquiry that is more accessible and have the potential to be adapted to high-throughput screening endeavors. These models allow for the development of microenvironments that closely mimic human mammary gland composition and can replicate a specific disease condition.

Healthy breast tissue consists of acini and ducts embedded in the ECM. The acini are highly organized structures, which contain a central lumen lined with polarized luminal epithelial cells. These luminal cells are surrounded by an outer layer of myoepithelial cells [116]. Both luminal and myoepithelial cells directly contact the BM [117], which is essential for maintaining epithelial cell polarity [82]. The breast ECM is typically composed of collagen I (the most abundant ECM component), laminin, collagen IV, fibronectin, and other proteins [118]. Its composition, density, and stiffness influence cell function and phenotype [119] while also regulating cell migration and positioning [120]. Breast epithelial cells in 2D monolayer culture (i.e., cells grown directly on plastic) lack environmental cues and thus do not form organized ducts or acini. By contrast, these structures are formed in 3D culture environments where cells at the center of the forming lumen are selectively cleared via apoptosis [105,121]. This process is closely regulated by the ECM [122].

Bi-directional cross talk was shown to exist between integrin and EGFR signaling pathways using 3D culture systems—a finding previously overlooked using 2D systems [106]. Studies of breast cancer cell lines cultivated in 3D matrices have accurately revealed connections between morphology, gene/protein expression, and invasive characteristics [123,124]. Bissell and colleagues have shown that the ECM affects chromatin structure and gene expression [125]. These findings underscore the importance of 3D culture models and their relevance to mammary gland biology under normal and pathological conditions.

## 4. Tumor-on-Chip Models: An Emerging 3D Technology to Study DCIS Transition

Organotypic tumor-on-chip models are defined as micro-fabricated cell cultures that combine microfluidics with 3D culture technology for the purpose of mimicking the complex characteristics of organs [126]. These model systems enable the investigation of complex interactions between cells and their microenvironment with real-time imaging capabilities. Many aspects of cellular physiology, such as morphology, polarity, cell adhesion, and cell–cell communication, are already widely studied in 3D reconstituted basement membrane (rBM) overlay systems (as described in [127,128]). Investigation of these physiological processes can be done using tumor-on-chip systems. Dynamic tissue characteristics such as oxygen gradients [129], shear stress [130], fluid gradients [131], and drug toxicity [132] can be interrogated. Due to their modular nature and adaptability for high-throughput screenings, tumor-on-chip models are increasingly being adopted in breast cancer research.

### 4.1. Microfluidics

Microfluidics is a technique that enables the control and analysis of fluids or nanobioparticles in microscale channels or structures [133]. In addition, they can also incorporate vascular and spatial control over fluids in micrometer-sized channels [134]. A variety of tumor-on-chip models that employ microfluidics are being developed to simulate DCIS and study the factors involved in its transition to invasive cancer. The majority of these, which are referenced here, incorporate well-established cell lines (see Table 1).

In an initial study, Sung and colleagues examined the spatial and temporal effects of cellular proximity in the microenvironment on DCIS transition [75]. In their microfluidic model, the group revealed that the distance between cancer cells and fibroblasts played a role in transition, and that the influence of CAFs may in part be due to the release of factors such as cytokines. They showed that, when cultured within 0.5–1.5 mm of fibroblasts, DCIS cells exhibited morphological changes. Furthermore, cell–cell contact with fibroblasts allowed DCIS to become invasive. This observation suggests that the secretion of signaling proteins resulted in DCIS invasion. Using this microfluidic model, Sung et al. were also able to evaluate changes in morphology by analyzing circularity, roundness, aspect ratio, collagen IV localization, and E-cadherin expression. Next, they compared their in vitro results to H&E staining of xenograft lesions in mice (performed simultaneously for validation). The morphology of DCIS clusters was observed by imaging filamentous actin (F-actin) protrusions via phalloidin staining [75]. F-actin organization has been described in tumor cell motility [152]. E-cadherin is an important component of cell–cell junctions, and its loss is a well-known characteristic of the invasive phenotype [153]. Here, loss of E-cadherin was observed in the invasive clusters. Collagen structure was also examined via second harmonic generation imaging in both separate compartments [115]. These were assayed to confirm the invasive phenotype in DCIS. Where the non-invasive DCIS clusters had consistent E-cadherin staining and rounded morphology, clusters co-cultured with fibroblasts showed a breakdown of collagen IV and partial loss of E-cadherin [115]. Thus, previously validated cellular components can be assayed using this microfluidic system.

A microfluidic model was designed to create physiologically relevant lumen structures. Their focus on constructing a physiologically relevant lumen is important because the geometry and spatiotemporal aspects of the lumen exert a profound influence on cellular behavior [75,154]. Here, the MCF10 progression series ([137] and Table 1) used a double-layered ECM hydrogel consisting of a Matrigel^TM^ inner layer surrounded by an outer layer of collagen I. The Matrigel^TM^ modeled the basement membrane, while two of the MCF10 lines were used to populate the lumen. MCF10A (a model of non-transformed epithelial cells) lined the lumen, while MCF10.DCIS was used to fill it, thus emulating human DCIS [75]. This model also demonstrated the ability to optimally image the components of adherens junctions between neighboring cells (via E-cadherin staining), as well as replicate proper apico-basal polarity (via the presence of GM130 and laminin 5), both of which are essential for normal epithelial structure. Thus, the value of this model is that it faithfully recapitulates key structures and functions of human DCIS.

### 4.2. Breast Cancer-on-a-Chip

Choi and colleagues have also illustrated a novel tumor-on-chip application [155]. Utilizing a co-culture of primary human fibroblasts and the cell lines HMT-3522 (a model of non-transformed breast epithelium) and MCF10.DCIS, they developed a system that replicated the architecture of DCIS. Their design consisted of a compartmentalized microdevice with upper and lower channels separated by a thin ECM-derived membrane. In the upper channel, DCIS spheroids were embedded in the mammary epithelial layer and supplied with a continuous flow of culture media. The lower channel (below the membrane) contained fibroblasts in a stromal layer, which was perfused with media to mimic the vascular compartment of mammary stromal capillaries. This system allowed for a 1-week co-culture, with over 85% cell viability. The authors also tested their system as a drug screening platform using paclitaxel as a test candidate. Choi et al. found that the treatment of MCF10.DCIS and HMT-3522 with paclitaxel caused a statistically significant increase in toxicity and reduction in tumor growth [155].

### 4.3. L-TumorChip

The L-TumorChip system was developed as a high-throughput tumor-on-chip platform to study breast tumor stromal interactions and drug pharmacokinetics [156]. This system incorporates a three-layered microfluidic platform that integrates tumor vasculature and tumor-stromal microenvironment with high-throughput screening ability. L-TumorChip has been used to investigate the influence of different stromal cells, CAFs, and mesenchymal stem cells on cancer cell growth responses to doxorubicin treatment. While the presence of CAFs delayed drug pharmacokinetics, apoptosis—indicated by caspase-3 activity—was increased in the presence of normal fibroblasts.

### 4.4. Microfluidic IDC-on-Chip to Study Epithelial-Endothelial Migration

Another tumor-on-chip system was specifically developed to investigate epithelial migration toward an endothelial lumen, and vice versa, in breast cancer. Here, they constructed two parallel lumens to characterize the cross-talk and co-migration behaviors of endothelial and epithelial cells [157]. This has been shown in tumor spheroids of other cancer cells [158,159] and other 3D systems [160]. The interaction between epithelial and endothelial cells is crucial for cancer progression, including DCIS. This is supported by studies recognizing that angiogenesis might possibly occur during the premalignant stage in cancers, including DCIS [161], due to increasing VEGF concentrations and other factors [162] and may be a predictor for invasive DCIS recurrence [163]. This aspect of DCIS progression is not well understood. Devadas and colleagues utilized the cell lines MCF10A, MCF-7 (ER+/PR+ breast cancer), and MDA-MB-231 (triple-negative breast cancer) to study migration behavior with human umbilical vein endothelial cells [157]. Migratory differences were observed in the three cell lines, with MCF10A exhibiting the least and MDA-MB-231 cells exhibiting a significant amount compared to MCF-7 cells.

### 4.5. Mammary Duct Model Capturing Matrix Mechanics

As mentioned earlier, the stiffness of the extracellular matrix influences normal and malignant cellular physiology. In certain aggressive breast cancers, increased stiffness corresponds to collagen linearization and increased deposition, leading to enhanced immune-cell infiltration [164]. Leveraging this information, Kulwatno and colleagues developed a microfluidic model that focuses on adjusting the stiffness and morphological properties of the matrix [165]. Using MCF10A, MCF10.DCIS, MCF10.CA1 (a model of invasive breast cancer), and MDA-MB-231 cells, they assessed breast cancer progression to invasion by focusing on the influences of matrix mechanics on DCIS progression and investigating collagen concentration-dependent behaviors in all three cell lines. Increased collagen concentration (stiffness) decreased the number of invaded cells of the MCF10.CA1 and MDA-MB-231 cell lines. MCF-7 cells formed smaller clusters in the ECM, with a lower collagen concentration compared to ECM with a higher collagen concentration. This model demonstrates the potential to capture the morphological characteristics of breast cancer development and to study the effects of tumor cell–ECM interactions.

### 4.6. Microfluidic Platform for Tumor Spheroid Invasion

Microfluidic models have been employed as tools to unveil the critical role of cell–cell adhesion in tumor invasion. Work by Huang and colleagues [166] investigated interstitial flow as a modulator on tumor invasion by redistributing signaling molecules within the tumor microenvironment [167]. This is important because it has been shown that tumor migration can be guided when interstitial flow facilitates secreted chemokine gradients via chemotaxis [168] and can activate integrins on the cell surface, eventually modulating cell migration on a 3D cellular matrix [130]. This work is in alignment with previous studies from our laboratory in which we have shown that paracrine signaling between DCIS and CAFs acts as an important factor in the transition of DCIS to invasive breast cancer [36].

Tumor-on-chip systems can also be used to assess and quantify the invasive potential of breast cancer cells [169]. Here, three different cell lines were used: MCF-7, MDA-MB-231, and SUM 159. The results show that while MDA-MB-231 and SUM 159 cells invaded the surrounding matrix, MCF-7 did not. The extent of the local invasion of cells was quantified via Image J analysis.

### 4.7. Microfluidic DCIS Model to Study Tumor Metabolism

The process of epithelial–mesenchymal transition requires a change in cellular metabolism, which itself has been implicated as a key player in the evolution to a malignant phenotype [170,171]. Changes in tumor metabolism include hypoxia, nutrient starvation, and waste product accumulation [172]. Additionally, adaptive mechanisms include niche construction due to changes in acidosis [173,174]. Microfluidics can be used to characterize these changes in the TME—including hypoxia and nutrient starvation—in a spatiotemporal manner. In a microfluidic model developed by Ayuso and colleagues [175], MCF10A and MCF10.DCIS cells were used to construct and fill a mammary duct, respectively. DCIS cell behavior was characterized by NMR spectroscopy and multi-photon optical metabolic imaging in addition to RT-qPCR and confocal microscopy. The results suggested that, compared to MCF10A cells, MCF10.DCIS cells have a faster oxygen metabolism that leads to hypoxia in the center of the lumen. DCIS cells were found to exhibit increased glycolysis via increased lactate levels and decreased glucose and pyruvate levels. Metabolic set enrichment analysis revealed 19 metabolic pathways that were altered between the DCIS and non-transformed mammary duct models, including pathways that were associated with rapid proliferation, as well as carbohydrate, nucleotide, and amino acid metabolism. NMR metabolomics analysis also revealed significant differences in metabolite concentrations and pathways between MCF10A and MCF10.DCIS cells. Optical metabolic imaging studies performed during the invasion process revealed that MCF10.DCIS cells had a higher redox ratio, consistent with a faster glycolytic metabolism [175].

### 4.8. MAME Model and TAME 3D Cell Culture Device

**Figure 1:**
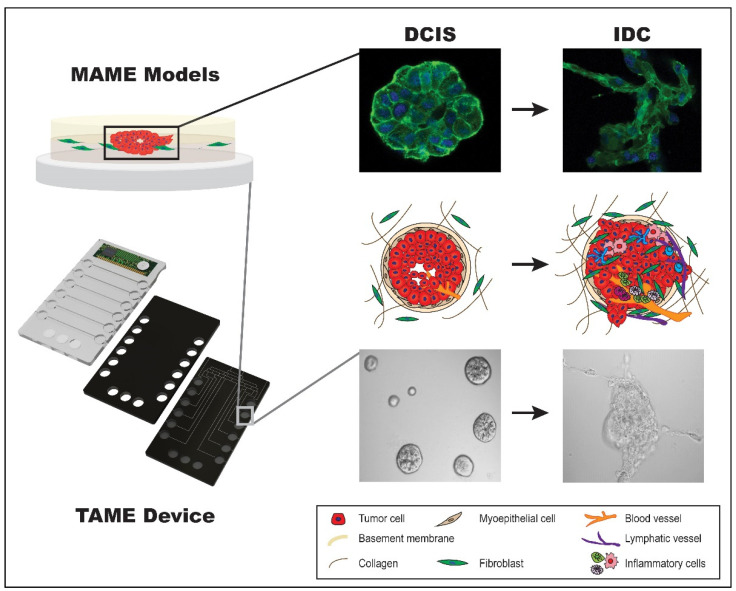
MAME Model and TAME Device. Our MAME models are 3D rBM overlay models designed to recapitulate the in vivo breast ductal landscape. Cells (one or more cell types) are seeded and allowed to grow for a number of days, and the resulting 3D structures are harvested and subjected to downstream applications such as mRNA/protein expression and immunofluorescence to study alterations in various cellular components, such as F-actin, during DCIS transition. Our TAME device incorporates multiple wells that represent our original MAME model and is applied to emulate paracrine signaling. Formation of structures as a result of monoculture or co-cultures are subject to downstream applications, including imaging [66,128,170].

Our interest and overarching goals are to develop models that recapitulate the architecture of pre-invasive breast lesions to study their progression to an invasive phenotype. We have developed 3D rBM overlay models, termed MAME (mammary architecture and microenvironment engineering) to create single or co-cultures in a 3D microenvironment [128]. Our 3D MAME cultures (Figure 1) are designed with Cultrex^TM^ (an rBM). Cell culture dishes are coated with 100% Cultrex^TM^ and cells are added on top and allowed to adhere. Once adhered, the overlay (2% Cultrex^TM^ in culture media) is added to the cells.

Our 3D models allow us to perform live imaging, functional live assays, and biochemical and immunohistochemical analyses [128]. We have used MAME models to delineate the molecular factors involved in the progression of DCIS to IDC. For example, we used whole transcriptome sequencing by mRNA-Seq for differential transcript expression profiling from the 3D rBM overlay cultures of DCIS models (MCF10.DCIS, SUM 102, and SUM 225) and parallel cultures of non-tumorigenic MCF10A cells as a model of non-transformed human mammary epithelial cells. This revealed that, out of 157 differentially expressed genes between the three DCIS lines and MCF10A cells, 63 were upregulated, within 244 promoter loci [176]. This screening and subsequent analysis resulted in three studies that delineated the roles of Aldehyde Dehydrogenase Isoform 5A1 (*ALDH5A1*) [176], Rap1Gap (*RAP1GAP*) [170], and Sprouty4 (*SPRY4*) [177] in DCIS progression. These three genes were viewed as strong candidates for further investigation based on Genomatix and Ingenuity Pathway analyses, which revealed significant enrichment (336-fold). *ALDH5A1*, which encodes an enzyme involved in mitochondrial glutamate metabolism, was found to be overexpressed in all three DCIS models. Treatment with disulfiram and valproic acid inhibited this enzyme and the net proliferation of DCIS cells [176], making it a potential molecular target.

**Figure 2:**
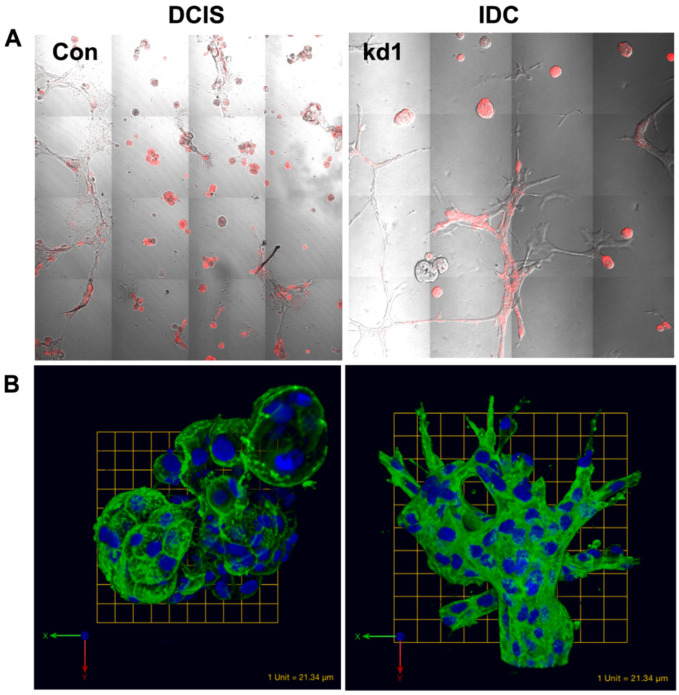
Application of MAME 3D culture model to evaluate changes in structural and cellular morphology after gene silencing. (**A**) Knockdown of Rap1Gap, a suppressor of DCIS invasion in DCIS cells grown in rBM shows the appearance of outgrowths and dramatic changes in cellular morphology. MCF10.DCIS cells were stably transfected with pSIREN dsRed shRNA expression vector to knockdown Rap1Gap. Cells were grown on rBM for 8 days and imaged live. Differential interference contrast imaged at 20X magnification with fluorescent overlay lentiviral control MCF10.DCIS and invasive MCF10.DCIS (kd1, knockdown of Rap1Gap). (**B**) Immunofluorescence for the evaluation of cellular characteristics in 3D. MCF10.DCIS cells were stably transfected with pSIREN dsRed shRNA expression vector to knockdown Rap1Gap expression. Cells were grown in rBM for 8 days and fixed. Fluorescent probes for the detection of filamentous actin (F-actin, green) and nuclei (blue) in the lentiviral control and invasive MCF10.DCIS. Images are 3D reconstructions of multiple z-stacks of overlaid green and blue channels. Shah et al., 2018 [170].

We subsequently identified the GTPase activating protein Rap1Gap, encoded by the gene *RAP1GAP*, as a molecular switch to IDC. Rap1Gap has previously been identified as a tumor suppressor in a variety of cancers through the inhibition of cellular proliferation, migration, and invasion [178,179,180,181,182]. We showed that, in a 3D rBM overlay cell culture system, downregulation of Rap1Gap via RNAi in MCF10.DCIS cells led to increased activation of extracellular regulated kinase/mitogen-activated protein kinase (ERK/MAPK), the formation of outgrowths, and increased invasion [170]. Figure 2A shows differential interference contrast images of round, compact DCIS structures (left panel) cultured in 3D rBM when transfected with the dsRed reporter control vector (con; referenced in [170]) and the appearance of invasive outgrowths in the right panel as a result of silencing Rap1Gap (kd1). The protruding outgrowths are indicative of extensive cytoskeletal reorganization in DCIS cells, assayed via the immunofluorescence of F-actin (see Figure 2B). Thus, we show how MAME cultures can be used to evaluate the molecular activity of potential tumor suppressors, including alterations in cytoskeletal organization via immunofluorescence in cells during DCIS transition.

The confirmation of Rap1Gap as a molecular switch from DCIS to IDC led to the recent investigation of the role of *SPRY4*. This gene encodes Sprouty4, a known modulator of ERK/MAPK signaling that is stimulated by receptor tyrosine kinase activity [183]. We have shown that the overexpression of Sprouty4 in the IDC cell line MCF10.CA1d suppresses ERK/MAPK phosphorylation, reverses their aggressive phenotype via the restoration of E-cadherin, reduces invasion, and reduces cellular proteolysis [177], a factor in the transition of DCIS to IDC [184].

**Figure 3:**
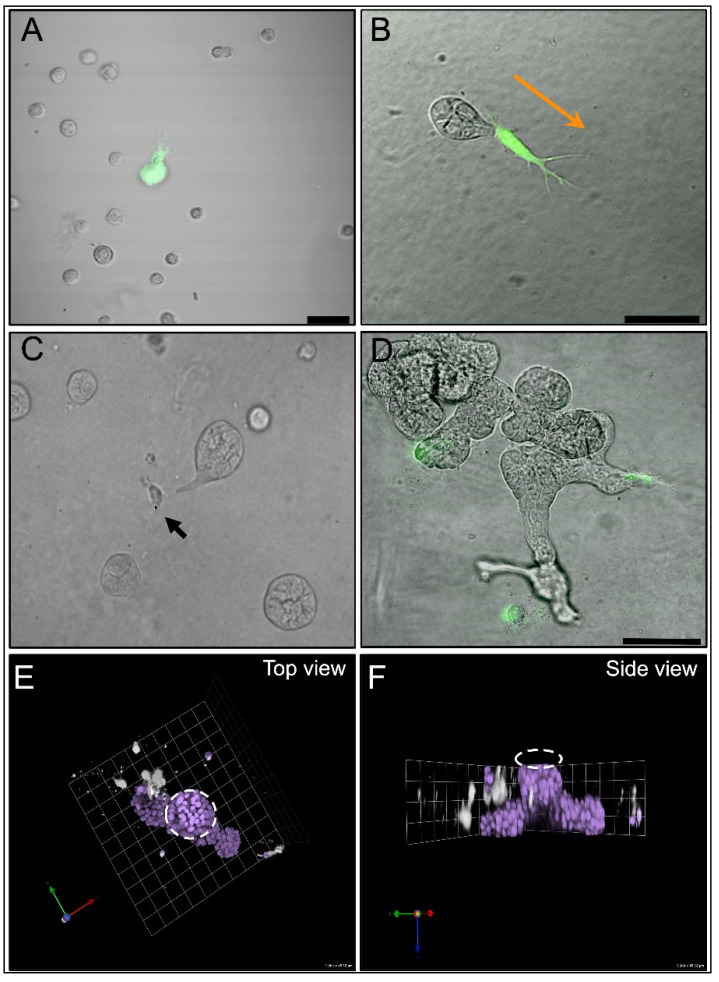
DCIS cells migrate preferentially towards CAFs in MAME co-culture. Representative differential interference contrast/fluorescent overlay image of MCF10.DCIS cells (unlabeled) with CAF40TKi fibroblasts (green) 24 h after seeding (**A**) and on day 3 (**B**). Scale bar, 50 µm. Orange arrow indicates migration direction. (**C**) Video snapshot of live cell imaging between days 3 and 4 shows a tumor spheroid attached to a single fibroblast (Arrow). (**D**) At day 7, tumor cells have proliferated and formed protruding structures, which are connected to fibroblasts. Scale bar, 100 µm. (**E**) Representative 3D reconstructed image showing aerial view (**E**) and side view (**F**) of MAME MCF10.DCIS:CAF co-culture. A primary MCF10.DCIS spheroid (dashed white circle) can be seen with lateral protrusions towards fibroblasts (CFSE-labeled and pseudo-colored white). Nuclei are stained with DAPI (purple). 1 Unit = 45 microns. Osuala et al., 2015 [36].

In addition to delineating the molecular factors that promote DCIS transition, we have also used 3D MAME co-culture models to mimic the tumor microenvironment. In particular, we investigated the role of the pro-inflammatory cytokine interleukin 6 (IL-6)—which is secreted by CAFs—in the progression of DCIS to IDC [36]. In CAF:MCF10.DCIS co-cultures, we evaluated cell–cell interactions, spatial organization, and motility via confocal microscopy. We observed that MCF10.DCIS spheroids formed attachments to CAFs and that the CAFs were located at the invasive edges of tumor spheroids (Figure 3). We also found that CAFs and MCF10.DCIS cells utilized cathepsin B to degrade the ECM. These studies revealed that CAFs secrete high levels of IL-6, which promoted DCIS cell proliferation and motility.

In a related study, we found that CAFs, when co-cultured with DCIS cells, induced the secretion of granulocyte macrophage-colony stimulating factor (GM-CSF) into the culture media. This induction of GM-CSF was associated with the activation of STAT3 in tumor cells and subsequent upregulation of GM-CSF gene expression [185]. Here we utilized the TAME (Tissue Architecture and Microenvironment Engineering) microphysiological systems device, a 3D cell culture device designed with capabilities including (1) use in live-cell imaging experiments for spatial–temporal evaluation of cell behavior, (2) perfusion system ready for the collection of secretomes, and (3) the ability to monitor culture microenvironment pH [184,186]. We further detailed a pro-inflammatory and pro-survival symbiotic feedback loop between tumor cells and CAFs [66,185].

We have also used MAME models to delineate the interactions between myoepithelial cells and CAFs within the DCIS microenvironment and the mechanism behind their tumor-suppressive activities. We have shown that myoepithelial cells reduce the dysplastic phenotype of DCIS and inhibit CAF-induced ECM proteolysis and invasion of DCIS structures [84]. This work suggests that the invasive transition of DCIS occurs via an increase in urokinase plasminogen activator secretion and proteolysis. The effects of myoepithelial cells supersede the tumor-promoting effects of CAFs by blocking IL-6 signaling pathways. Our work is an example of how 3D culture can be utilized to study the intercellular dynamics in the tumor microenvironment and various molecular factors in the transition of DCIS to IDC [84].

We also examined the expression of vimentin, which under normal conditions maintains the normal morphology of cells and acts as an anchor in the nucleus and organelles [187] and is involved in the development of the mammary gland [188]. In terms of cancer, vimentin has been reported to regulate EMT, implicating its role in the development of tumors [189]. Vimentin has also been shown to play an important role in the promotion of cancer cell migration [188]. Other studies have shown that its expression in triple-negative breast cancers was significantly associated with histological grade, lymph node invasion, and especially with the absence of ER expression [190]. We found that some CAFs expressed vimentin and IL-6 and may represent a subset of the CAF population [36]. This is an area of interest for future studies.

### 4.9. 3D Culture Platform Limitations

Three-dimensional cell culture platforms provide biological advantages over traditional 2D cell culture and a financial and ethical benefit over the use of animal models. However, 3D cell culture methodologies have limitations. The setup and maintenance of 3D cell cultures is more time consuming and requires a greater level of cell culture expertise than traditional 2D cell cultures; 3D cultures are inherently more complex, and the absence of a standardized protocol limits the collective adoption of the technology by researchers.

The ideal goal of 3D cell cultures is to develop in vitro organoid systems that mimic the in vivo state. The 3D culture methodologies published to date are getting closer and closer to achieving this goal [191,192,193] as many cancer models can accommodate up to three different cell types [194]. There is still much work to do because in vivo cancer tissue systems may contain more than seven different cell types (tumor cell, lymphocytes, macrophages, fibroblasts, neutrophils, adipocytes, endothelial cells, and benign epithelium) [195]. Microfluidic-based models offer an advantage in that they can be adapted to co-culture experiments, allowing for the interrogation of interstitial fluid flow/vasculature in or around the culture [196].

There is also a significant cost to 3D cell cultures as compared to 2D cultures. The extra time needed for 3D culture setup is a key driver of costs, along with the cost of the extracellular matrix (which in most cases is the most expensive single-use consumable). Another major limitation of 3D cell culture is its scalability, particularly regarding drug discovery. For example, to test thousands of available drugs, millions of cells are traditionally needed. This is relatively easy and fast to achieve with 2D cultures, whereas this is not feasible using current 3D culture technologies [197]. While many novel platforms are being developed to address the scalability of 3D models, they are far less robust than traditional 2D cell cultures [198]. In addition to the technical challenges of scalability, there are scientific hurdles to overcome, such as heterogeneity between 3D spheroids, genetic drift in long-term 3D cultures, and reproducibility of results.

Standardization and clinical validation are also major hurdles that 3D culture technology must surmount before it is seen as a viable alternative to animal models. The scientific community will need to coalesce around a small group of 3D methodologies that are suitable for all cell types and are not too complex or expensive. This standardization will facilitate the necessary studies needed for clinical validation. Although the costs of using 3D cell culture methodologies are high, when measured against the costs associated with animal models it is clear that the technology is worth pursuing.

## 5. Conclusions

Over the past decade, tumor heterogeneity has been shown to extend far beyond tumor cells alone, including many components of the TME, and has emerged as a risk factor that drives tumor progression. The heterogeneity in the DCIS TME is thought to contribute to the uncertainty of which tumors are likely to progress versus those that will remain indolent. Understanding the nature and regulation of interactions within the TME is a necessary step in revealing reliable biomarkers for the development of novel anti-cancer therapies. Heterogeneity in DCIS and its transition [5,39] partly manifests in the variation seen in clinicopathological features such as biological grade, ER/PR status, HER2 status [199,200], and luminal and basal subtypes [201,202]. This heterogeneity also includes high variability in the composition of the TME, i.e., the density and spatial distribution of stromal cells [39,203,204]. Moving forward, studies using 3D culture models of DCIS should aim to decode the interactive signaling between DCIS cells and their neighbors in the TME. Having the capability to understand this crosstalk will deepen our understanding of DCIS cancer biology and its mechanistic paths to invasive breast cancer.

## Figures and Tables

**Table 1:** Breast cell lines used in tumor-on-chip models.

Non-Transformed Lines	Origin	ER/PR/HER2 Status	Model
HMT-3522-S1	Fibrocystic breast tissue [135]	–/–/– [136]	Epithelium
MCF10A	Fibrocystic breast tissue [137]	–/–/– [138]	Epithelium
**DCIS Lines**	**Origin**	**ER/PR/HER2** **Status**	**Model**
MCF10.DCIS.com	Progression from MCF10A [139]	–/–/– [110,140]	DCIS
SUM 102	Surgically resected DCIS with microinvasion [141]	–/–/– [136]	DCIS with microinvasion
SUM 225	DCIS chest wall recurrence [142]	–/–/+ [110,136]	DCIS
21NT	Infiltrating intraductal carcinoma [143]	–/–/+ [136]	DCIS
hDCIS.01	Hyperplastic columnar cell hyperplasia [144]	–/–/– [145]	DCIS
FSK-H7	HER2+ DCIS [110]	–/–/+ [110]	DCIS
ETCC006	Pre-invasive DCIS [146]	U [146,147]	DCIS
**Transformed Lines**	**Origin**	**ER/PR/HER2 Status**	**Model**
MCF-7	Pleural effusion from metastatic carcinoma [148]	+/+/– [136,138]	Luminal A invasive carcinoma
MCF10.CA1d	Progression from MCF10A [149]	–/–/– [140,150]	Basal-like invasive carcinoma
SUM 159	Anaplastic carcinoma of the breast [141]	–/–/– [136]	Basal-like invasive carcinoma
MDA-MB-231	Pleural effusion from patient with metastatic adenocarcinoma [151]	–/–/– [136,138]	Basal-like invasive carcinoma

U: Unknown or conflicting reports in the literature.

## Data Availability

The original contributions presented in this study are included in the article. Further inquiries can be directed to the corresponding author(s).
